# Estrogen Inhibits Renal Cell Carcinoma Cell Progression through Estrogen Receptor-β Activation

**DOI:** 10.1371/journal.pone.0056667

**Published:** 2013-02-27

**Authors:** Cheng-Ping Yu, Jar-Yi Ho, Yi-Ting Huang, Tai-Lung Cha, Guang-Huan Sun, Dah-Shyong Yu, Fung-Wei Chang, Shu-Pin Chen, Ren-Jun Hsu

**Affiliations:** 1 Biobank Management Center of Tri-Service General Hospital, National Defense Medical Center, Taipei, Taiwan; 2 Graduate Institute of Pathology and Parasitology, Tri-Service General Hospital, National Defense Medical Center, Taipei, Taiwan; 3 Graduate Institutes of Life Sciences, National Defense Medical Center, Taipei, Taiwan; 4 Divisions of Urology, Tri-Service General Hospital, National Defense Medical Center, Taipei, Taiwan; 5 Department of Obstetrics & Gynecology, Tri-Service General Hospital, National Defense Medical Center, Taipei, Taiwan; 6 Children's Endocrine and Genetic Disciplines, Chang Gung Children's Hospital, Taoyuan, Taiwan, ROC; National Cancer Institute, United States of America

## Abstract

Renal cell carcinoma (RCC) originates in the lining of the proximal convoluted tubule and accounts for approximately 3% of adult malignancies. The RCC incidence rate increases annually and is twofold higher in males than in females. Female hormones such as estrogen may play important roles during RCC carcinogenesis and result in significantly different incidence rates between males and females. In this study, we found that estrogen receptor β (ERβ) was more highly expressed in RCC cell lines (A498, RCC-1, 786-O, ACHN, and Caki-1) than in breast cancer cell lines (MCF-7 and HBL-100); however, no androgen receptor (AR) or estrogen receptor α (ERα) could be detected by western blot. In addition, proliferation of RCC cell lines was significantly decreased after estrogen (17-β-estradiol, E2) treatment. Since ERβ had been documented to be a potential tumor suppressor gene, we hypothesized that estrogen activates ERβ tumor suppressive function, which leads to different RCC incidence rates between males and females. We found that estrogen treatment inhibited cell proliferation, migration, invasion, and increased apoptosis of 786-O (high endogenous ERβ), and ERβ siRNA-induced silencing attenuated the estrogen-induced effects. Otherwise, ectopic ERβ expression in A498 (low endogenous ERβ) increased estrogen sensitivity and thus inhibited cell proliferation, migration, invasion, and increased apoptosis. Analysis of the molecular mechanisms revealed that estrogen-activated ERβ not only remarkably reduced growth hormone downstream signaling activation of the AKT, ERK, and JAK signaling pathways but also increased apoptotic cascade activation. In conclusion, this study found that estrogen-activated ERβ acts as a tumor suppressor. It may explain the different RCC incidence rates between males and females. Furthermore, it implies that ERβ may be a useful prognostic marker for RCC progression and a novel developmental direction for RCC treatment improvement.

## Introduction

Estrogen is a female hormone secreted mainly by the ovaries to promote the development of the female reproductive system and the proliferation of the endometrium as part of the menstrual cycle. During the child-bearing period, estrogen shows periodic changes with fluctuating secretion. The functions of estrogen include the promotion of subcutaneous fat accumulation and mammary gland proliferation, water and sodium retention and calcium deposition, avoidance of coronary atherosclerosis, and prevention of osteoporosis and Alzheimer's disease. The bioeffect of estrogen is evident through binding to estrogen receptors (ERs) and subsequent regulation of the transcription and activation of downstream genes. There are two subtypes of ERs, namely estrogen receptor α (ERα) and estrogen receptor β (ERβ). Distribution of ERα and ERβ varies in different tissue types [1].

The correlation between ERα and breast cancer has been extensively studied and proven. However, the actual molecular mechanism of ERβ is still unclear. ERβ is the second type of ER. Although the structures of ERα and ERβ are similar, their histological distributions and biological functions are not the same. Previous studies have shown that ERβ expression in cancerous cells was lower than that in normal cells [2]; other studies have also demonstrated that ERβ decreases proliferation and induces apoptosis. Thus, it was deduced that ERβ may play a role as a tumor suppressor in carcinogenesis [3], [4].

Renal cell carcinoma (RCC) is the third leading cause of death among urological tumors (85% of the adult kidney cancer), accounting for 3% of adult malignancies [5]. The pathology of RCC includes the following: (1) clear cell carcinoma, the most common type of RCC, accounting for 70–80% of RCC; (2) papillary carcinoma, characterized by papillary growth and accounting for 10–15% of RCC; and (3) chromophobe RCC, accounting for 5% of RCC [5], [6]. According to the latest statistics from the U.S, EU, and Taiwan, the incidence of RCC is increasing, and the age of occurrence is between 50 and 70 years [7]–[10]. The incidence in males is higher than that in females, with a ratio of 2∶1; however, the cause for the difference in the male-to-female ratio is unclear. There are various proposed risk factors for this ratio [11]–[16], but the increase in RCC incidence in females after hysterectomy drew attention. The decrease in estrogen after hysterectomy may be one of the causes of this increased risk.

Therefore, we hypothesize that estrogen inhibits RCC carcinogenesis and progression and that there might be a biological effect of estrogen on RCC. We aim to observe the biological and clinical effects of ERα and ERβ in RCC. In this study, we found that estrogen inhibited the proliferation, migration, and invasion of RCC cells and increased RCC apoptosis. With respect to the molecular mechanisms, estrogen, through ERβ, affected the expression of growth factor-related downstream genes (p-AKT, p-ERK, and p-JAK) and apoptotic genes. These results illustrated that ERβ suppresses tumor growth, providing a possible explanation for the difference in RCC incidence between males and females. After investigating the molecular mechanisms, ERβ, as a bioindicator, may provide a new option for the prediction, progression, and treatment of RCC.

## Materials and Methods

### Ethics statement

All subjects signed a written informed consent form. All study procedures were approved by the Institutional Review Board of the Tri-Service General Hospital, National Defense Medical Center (TSGH-IRB-098-05-221 and TSGH-IRB-099-05-165).

### Cell culture and chemicals

The human RCC cell lines 786-O, A498, ACHN, Caki-1, and RCC-1 and the human breast cancer cell lines MCF7 and HBL-100 were purchased from [Bioresource Collection and Research Center (BCRC)]. The cells were maintained with DMEM or RPMI media containing 10% FBS in a cell incubator (37°C, 5% CO_2_). Human estrogen [β-Estradial] (Sigma-Aldrich) was dissolved in ethanol, and a 10 µM stock solution was prepared. The working concentration for estrogen was 10 nM.

### Analysis of the effect of estrogen on cell growth

MTT (3-(4,5-cimethylthiazol-2-yl)-2,5-diphenyl tetrazolium bromide) assay (MTT reagent, Bio Basic) was used to detect cell growth. Each well of a 96-well microplate contained approximately 2000 cells. After overnight culture, estrogen (final concentration 10 µl/ml) or ethanol (control) were added to these wells and cultured for 3 days to prepare a cell growth curve. For detection, 50 µl MTT reagent (2 mg/ml) was added and incubated at 37°C with 5% CO_2_ for 3 h. After removing the MTT reagent, 200 µl DMSO was added to the wells at room temperature for 10 min with gentle shaking. The reaction was detected using a microplate reader (Thermo Scientific) at 540 nm, and absorbance was used for preparation of a cell growth curve. Values for the treatments (estrogen or ethanol) were the OD average of 6 repeats.

### Cell transfection

To overexpress ERβ, a plasmid containing ERβ was constructed (pcDNA3.1-ERβ). A cell line with low ERβ expression (A498, see results) was used for ERβ overexpression. To knockdown ERβ expression, siRNA for ERβ (Stealth siRNAs HSS103378, HSS176622, HSS103380, Life Technologies) was induced in a 789-O cell line (high ERβ expression, see results). Transfection was performed with Lipofetamine 2000 reagent (Invitrogen). After culturing 1×10^6^ cells in a 6-cm culture dish for 8 h, the culture medium was removed and the cells were washed. The cells were further cultured in FBS-free media overnight at 37°C with 5% CO_2_. After mixing an appropriate amount of nucleic acid (pcDNA3.1-ERβ or ERβ siRNA) with 200 µl OPTI-MEM (GIBCO) and 6 µl Lipofetamine 2000 reagent with 200 µl OPTI-MEM (GIBCO) in a separate tube, the reactions were allowed to stand for 5 min at room temperature. The Lipofetamine 2000 reaction was added to the nucleic acid-containing tube, and the mixture was allowed to stand for 20 min at room temperature. The mixture was then added to the 6-cm culture dish for 8–12 h (37°C, 5% CO_2_). After incubation, the mixture was replaced with media containing FBS, and the cells were cultured at 37°C with 5% CO_2_ for 48 h. The transfected cells were collected for RNA or protein assays.

### Wound healing assay

The cells were seeded in 6-cm culture dishes and cultured overnight until they had reached 90% confluence. After cell adhesion, a pipette tip (300 µl) was used to draw a cell-free line (approximately 1 mm wide) on the dish. After removing the original media and washing with PBS, media containing 10 nM estrogen was added to the cells. The condition of wound healing was observed and photographed using a microscope at 0, 24, and 48 h.

### Migration and invasion assay

In the invasion assay, one layer of 2% Matrigel was used to cover the 24-well micropore polycarbonate membrane filter, and this step was omitted in the migration assay. The cells (2×10^4^) were seeded in the top chamber of the 24-well micropore polycarbonate membrane filter (8 µm) and cultured with FBS-free media at 37°C with 5% CO_2_ for 24 h (migration assay) or 48 h (invasion assay). The 24-well micropore polycarbonate membrane was then fixed with 70% formalin at room temperature for 30 min, followed by washing with PBS. The membrane was stained with crystal violet for 30 s and washed with water for 30 s. Cells that did not crawl over were removed with cotton swabs. The chamber was cut and placed on a glass slide by a clean scalpel, and mineral oil was added to the slide. The number of cells that passed through the polycarbonate membrane was counted under a microscope.

### Western blot

Subconfluent cells were washed with ice-cold PBS and lysed in RIPA lysis buffer (containing 1X protease inhibitor and an 1X phosphatase inhibitor). Protein concentrations were quantified by BCA protein assay (Pierce), and an equal concentration of lysate (30 or 50 µg/ml) was subjected to 8% SDS–PAGE and transferred to nitrocellulose membranes. The membranes were then blocked with 1% blocking buffer (1% BSA in TBS-T) before the primary antibody reaction at 4°C overnight. Secondary antibody reacted with the membrane at room temperature for 1 h. The proteins were visualized using HRP-conjugated secondary antibodies and ECL chemiluminescent reagent (Pierce) for western blots. The following primary antibodies were used: rabbit anti-ERα, rabbit anti-ERβ, rabbit anti-AR, rabbit anti-ERK1, rabbit anti-phospho-JNK2, rabbit anti-JNK2, rabbit anti-phospho-STAT3 (PY705), rabbit anti-STAT3, rabbit anti-MMP-9, rabbit anti-pro-caspase-3, rabbit anti-active-caspase-3, rabbit anti-Bcl-2, and rabbit anti-survivin (all from Epitomics); mouse-anti-β-actin (Thermo Scientific); mouse anti-V5-tag (AbD Serotec); mouse anti-phospho-ERK1/2 (E4) and rabbit anti-Bid (FL-195) (Santa Cruz Biotechnology); and rabbit anti-phospho-AKT (Ser473), rabbit anti-pan-AKT, rabbit anti-phospho-P70S6K (Thr389), rabbit anti-P70S6K, rabbit anti-phospho-GSK3 (Ser9), rabbit anti-GSK3, rabbit anti-phospho-PTEN (S380), rabbit anti-phospho-NFκB (p65), rabbit anti-NFκB, rabbit anti-caspase-8, rabbit anti- cleaved-caspase-8, rabbit anti-caspase-9, and rabbit anti-cleaved-caspase-9 (all from Cell Signaling Technology). HRP-conjugated secondary antibodies (rabbit anti-mouse and goat anti-rabbit) were from Jackson ImmunoResearch Laboratories.

### Reverse transcriptase–polymerase chain reaction (RT-PCR)

Total cellular RNA was isolated from subconfluent cells cultured in 6-cm dishes using Trizol® RNA Extraction Reagent following the manufacturers' instructions. The RNA concentration was determined using the NanoDrop UV spectrophotometer (Thermo Scientific). Reverse transcription was performed with SuperScript® III Reverse Transcriptase (Invitrogen®). Real-time PCR was performed with SYBR Green qPCR Master Mix (2X) (Fermentas) and measured on real-time PCR systems (Applied Biosystems StepOne™). CT values were reported relative to GAPDH RNA. Primers used were as follows: ERβ-5′-GTCAGGCATGCGAGTAACAA-3′, ERβ-3′- GGGAGCCCTCTTTGCTTTTA-5′; and GAPDH-5′-CCACTCCTCCACCTTTGAC-3′, GAPDH-3′-ACCCTGTTGCTGTAGCCA-5′. The primers were synthesized by MDBio Inc.

### Flow cytometry

Flow cytometry for analyzing the cell cycle (propidium iodide staining) was performed according to standard procedures. Single cell suspensions were prepared by harvesting cultured cells in 6-cm dishes and reacting them with propidium iodide (50 µg/ml containing 200 µg/ml RNase). The reaction was analyzed on a flow cytometer (BD Biosciences) for cell cycle detection.

### Immunohistochemistry (IHC)

Formalin-fixed, paraffin-embedded tissue was sectioned into 3-µm slices and placed on glass slides covered by 2% Absolve. After de-waxing (xylene) and rehydration (95% and 75% ethanol), the tissue was subjected to antigen retrieval in citrate buffer (pH 6) in a high-pressure oven. A Dako pen was used to draw a waterproof circle to ensure that the tissue was completely immersed in antibody reagents. Coloration was performed by the DAB method using the IHC kit (UltraVision Quanto Detection System HRP, Thermo Scientific) according to the manufacturers' instructions, and the tissue was counterstained with hematoxylin. The slices were mounted using mounting media and observed under microscopy.

### Statistics

The western blot results were quantified with Image J 1.46x software (http://rsb.info.nih.gov/ij/download.html) and those of immunohistochemistry were quantified with Aperio ImageScope and Spectrum Software ver. 10.0. All quantification values were continuous variables, and based on the number of groups, they were analyzed by Student's *t*-test or one-way ANOVA with the least significant difference (LSD) post-testing method to compare the average values.

Correlations between RCC occurrence and ERβ expression, age at diagnosis, and gender were analyzed by logistic regression (disease group n = 85; benign control group n = 118). The correlation between RCC prognosis and ERβ expression in the RCC disease group and interaction between various clinical prognosis factors was further analyzed. Survival analysis was first presented as a Kaplan–Meier survival curve coordinated with the log-rank test to assess the effect of ERβ expression on RCC survival. Furthermore, univariate analysis on the ERβ expression and various clinical prognosis factors was performed with the Cox hazard regression model, and factors showing significance were further evaluated with multivariate analysis. The correlation between the factors was assessed. Statistics were performed with SPSS 16.0 and Excel 2007, and the statistical significance was two-tailed and set at p<0.05 (*), p<0.01 (**), or p<0.001(***).

## Results

### Effect of estrogen on cell growth in RCC cell lines

**Figure 1:**
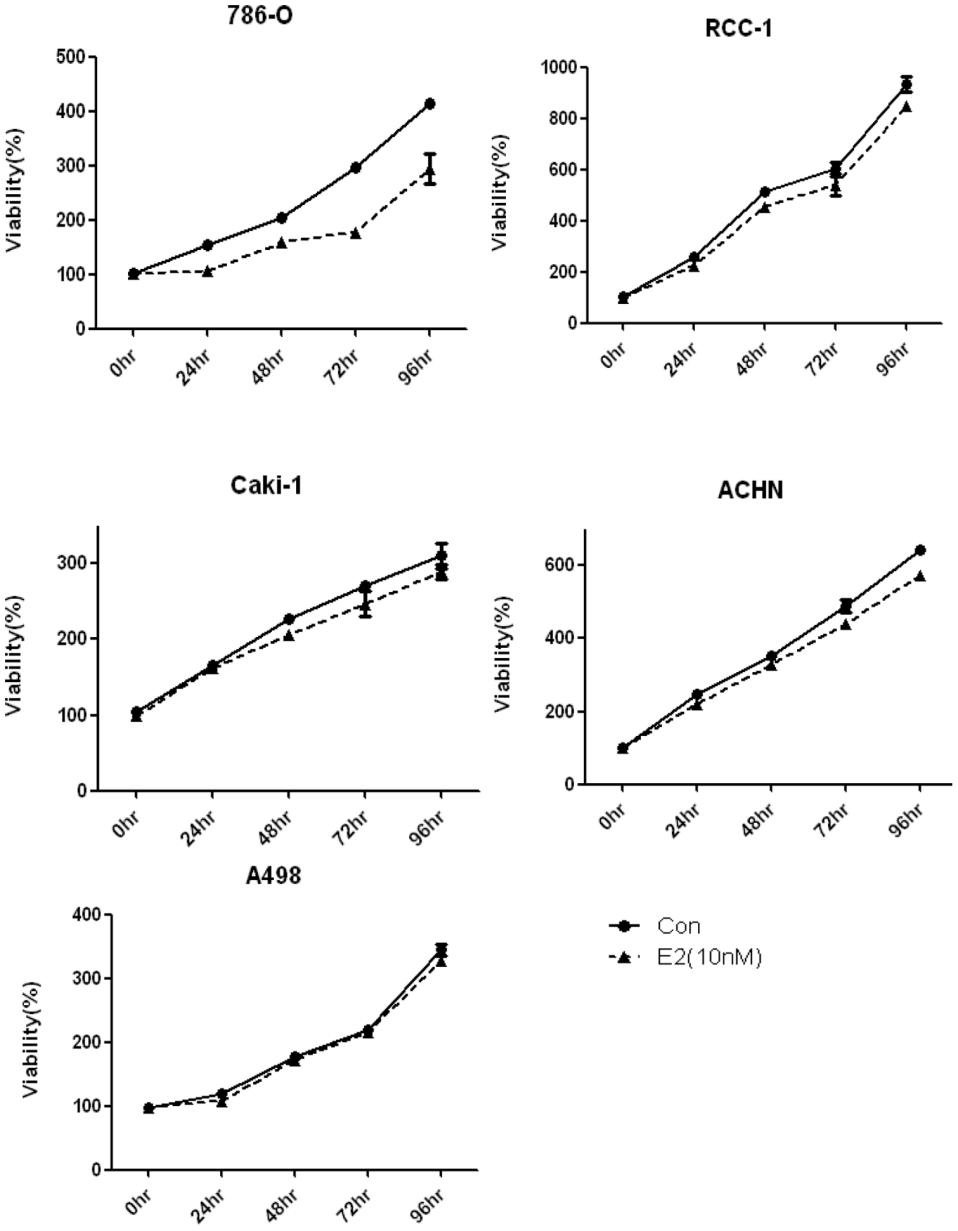
Effect of estrogen on cell growth in the RCC cell line. In the CC cell lines 786-O, RCC-1, Caki-1, and ACHN, cell growth slowed down in the cells treated with estrogen (10 nM) compared with the control (ethanol). Only A498 was not affected by estrogen treatment. The experiments were repeated at least three times.

The RCC cell lines 786-O, RCC-1, A498, Caki-1, and ACHN were cultured in 96-well microplates and stimulated with 10 nM estrogen or an equal amount of ethanol (control). After 0, 24, 48, 72, and 96 h, cell growth was analyzed with MTT assay. The results showed that except for A498, the growth rates of all cell lines were slower in cells stimulated with estrogen compared with those stimulated with the control. The effect of estrogen was strongest in the 786-O cell line (Figure 1). These results indicate that estrogen stimulation reduces cell growth in RCC cell lines.

### ERβ protein expression in RCC cell lines and RCC tissue

**Figure 2:**
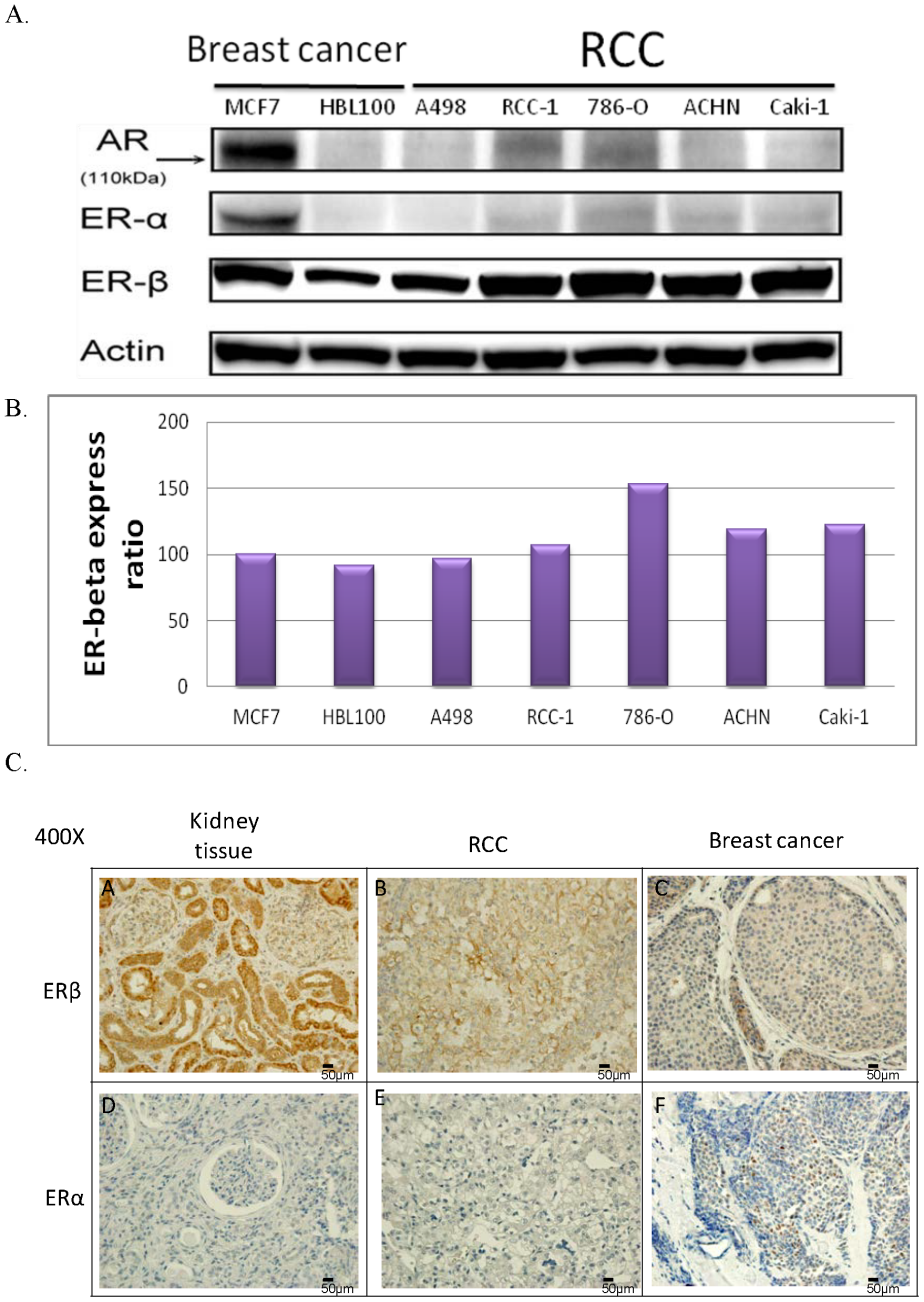
Expression of AR, ERα, and ERβ in breast cancer and RCC cell lines, and the expression of ERα and ERβ in kidney, RCC, and breast cancer tissue samples. (A) (B) In breast cancer cell lines, MCF-7 showed AR, ERα, and ERβ expression, while HBL100 showed no ERα expression and only low ERβ expression. In RCC cell lines, no AR expression was observed. Except A498, the RCC cell lines showed higher ERβ expression than the breast cancer cell lines. Expression of ERβ was the highest in 786-O. (C) IHC results for the observation of ERα and ERβ expression in kidney, RCC, and breast cancer tissue samples. (C-a) Cytoplasm and nuclei of the kidney tissue showed high expression of ERβ. (C-b) In RCC tissue, ERβ expression was mainly found in the cytoplasm. (C-c) Low expression of ERβ was observed in the cytoplasm of breast cancer tissue. (C-d) (C-e) No ERα expression was found in the kidney or RCC tissue. (C-f) ERα expressed in the nuclei of breast cancer tissue. Densitometry analyses for protein quantification were done using Image J 1.46x software (http://rsb.info.nih.gov/ij/download.html). The experiments were repeated at least three times. MCF7 expression was the baseline expression for the quantification comparison of ER<beta>. The results were analyzed with t-test.

In most RCC cell lines, cell growth is reduced with estrogen treatment. The main effectors of estrogen are ERα and β (ERβ). Moreover, the male/female hormones differ not only in estrogen levels but also in androgen levels. The main effector for androgen is the androgen receptor (AR). Therefore, protein expression of ERα, ERβ, and AR was observed in the RCC cell lines. Human breast cell lines served as controls, where MCF7 was ERα positive and HBL100 was ERα negative. Western blot results showed that all cell lines [MCF7, HBL100, and five RCC cell lines (786-O, RCC-1, A498, Caki-1, and ACHN)] had no AR or ERα protein expression. On the other hand, ERβ expression in the RCC cell lines 786-O, RCC-1, Caki-1, and ACHN was higher than in the breast cancer cell lines MCF7 and HBL100; however, ERβ expression in A498 was relatively lower. In particular, ERβ expression in 786-O was the highest (Figure 2A, 2B). This might provide the rationale that A498 did not respond to estrogen treatment while 786-O showed the most significant reaction.

To observe ERα and ERβ expressions in normal kidney, RCC, and breast cancer tissue, IHC was performed on formalin-fixed, paraffin-embedded tissue samples. The results showed that ERβ was expressed in both the nuclei and cytoplasm of normal kidney tissue (Figure 2C-a). In contrast, ERβ expression was lower in RCC tissue, with predominantly cytoplasmic staining (Figure 2C-b). In breast cancer tissue, ERβ expression was the lowest (Figure 2C-c). With respect to ERα, only breast cancer tissue showed ERα expression (Figure 2C-f), and normal kidney and RCC tissue showed no ERα expression (Figure 2C-d, 2C-e). These results were consistent with the western blot results. Thus, we deduced that in RCC, ERβ rather than ERα was expressed in both cell lines and tissue samples.

### Clinical validation experiments of ERβ and RCC

Using the case–control study method, 118 patients with benign kidney disease lesions and 85 patients with renal cell disease were selected through retrospective recruitment. The representative parts of the paraffin-embedded tissue were used to construct a tissue microarray, which was used for ERβ immunohistochemistry. The correlations between RCC and expression of ERβ, age at diagnosis, and gender were analyzed with logistic regression. The results of univariate analysis (Table 1) showed that individuals with low ERβ expression or higher age at diagnosis were more likely to develop RCC. However, the correlation between gender and RCC was not statistically significant. In multivariate analysis, results showed that individuals with lower ERβ expression still had a higher risk of RCC after age adjustment than those with high ERβ expression [relative risk (OR) = 0.059, 95% CI: 0.027–0.129].

**Table 1:** Correlation between the risk factors of RCC and the expression of ERβ.

Subject characteristics	RCC (n = 85)	BRD (n = 118)	Univariate		Multivariate	
			OR (95% CI)	p†	OR (95% CI)	p†
**ERβ expression** *						
Low	73	30	1 (ref)		1 (ref)	-
High	12	88	**0.056 (0.027–0.117)**	**<0.001**	**0.059 (0.027–0.129)**	**<0.001**
**Demographic factors**						
Age	60.74 ± 13.71	48.21 ± 17.95	**1.048 (1.028–1.068)**	**<0.001**	**1.047 (1.022–1.072)**	**<0.001**
Gender – Female	29	54	1 (ref)			
Male	56	64	1.629 (0.916–2.900)	0.097	-	-

* Categorized as low (≤mean) and high (>mean) was separated at ERβ >35% positivity (high) and ERβ ≤35% positivity (low).

† Based on the logistic regression model. Statistical significance (p<0.05) is shown in boldface. n.a.: not analyzed.

Abbrev: RCC: renal cell carcinoma, BRD: benign renal disease, OR: odds ratio, CI: confidence interval; ER: estrogen receptor.

**Figure 3:**
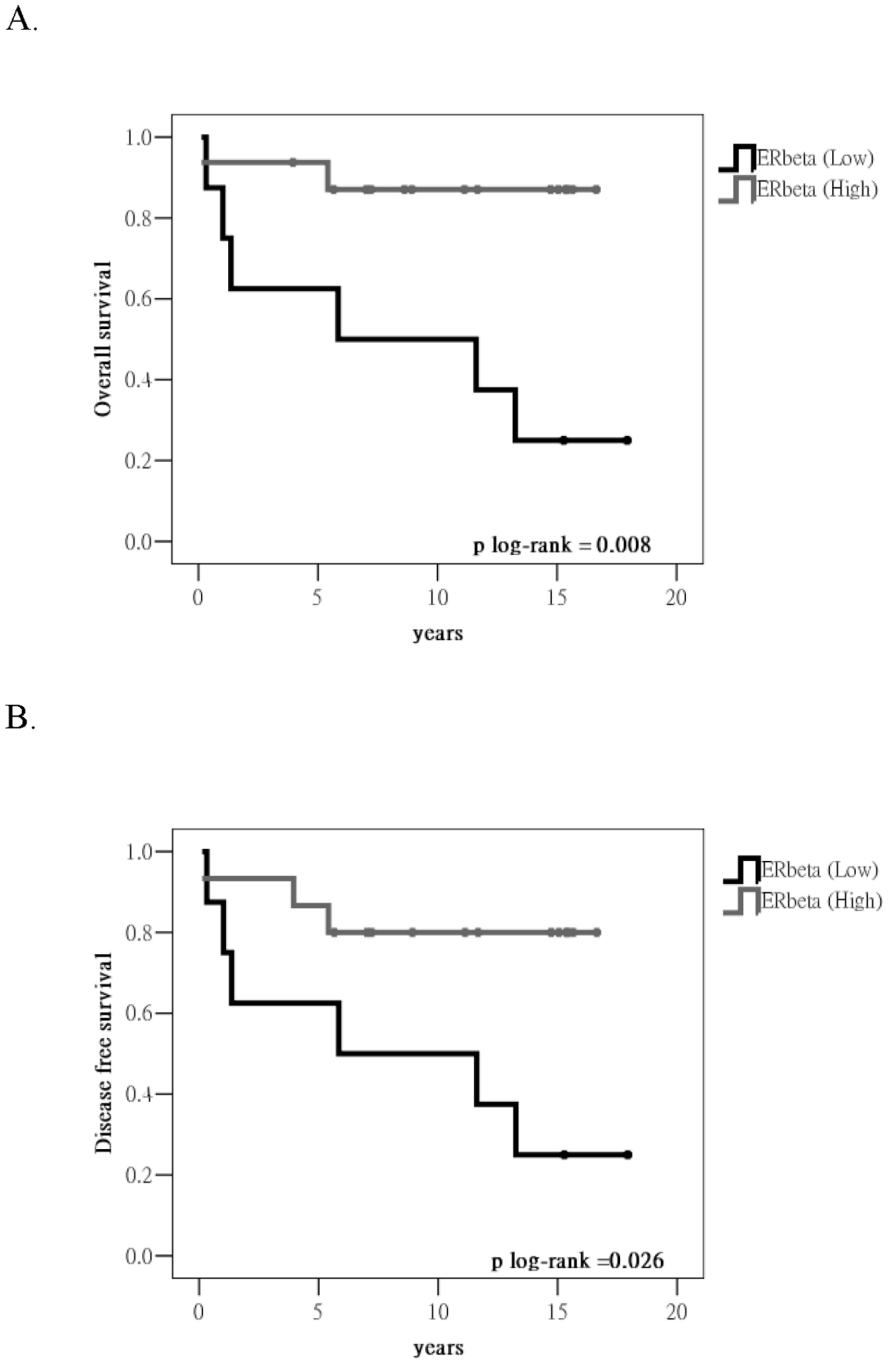
Kaplan–Meier survival curve: the correlation between ERβ expression and RCC prognosis, assessed by univariate survival analysis. (A) Correlation analysis of ERβ expression and the overall survival of RCC (B) Correlation analysis of ERβ expression and DFS of RCC.

In individuals with RCC, the correlation between ERβ expression and RCC prognosis was further analyzed. The survival curve for the cases with different levels of ERβ expression was drawn using the Kaplan–Meier method (Figure 3). Cases with low ERβ expression showed poorer prognosis. The log-rank test revealed that overall survival (OS) and disease-free survival (DFS) were statistically significant. Furthermore, the correlation between ERβ expression and various clinical prognosis factors was analyzed with the Cox hazard regression model (Table 2). Univariate analysis revealed that cases with low ERβ expression had poor prognosis, and OS and DFS were statistically significantly different. In addition, cases with higher clinical stages and pathological grades also showed poor prognosis. On assessment of DFS, males showed a slightly poorer prognosis than females, while other clinical prognosis factors showed no significant correlation with poor RCC prognosis. Multivariate analysis showed that the death rate in patients with higher ERβ expression was 1/10 that of patients with lower ERβ expression (OS: 0.118 times, DFS: 0.082 times; see Table 2). In addition, the death rate of patients of higher clinical stage (stage III–IV) was more than 3 times higher than that of patients with lower clinical stage (stage I–II) (OS: 3.595 times, DFS: 3.290 times; see Table 2). Moreover, ERβ expression and clinical stage were independent variables in multivariate survival analysis. That is, the expression of ERβ in renal tissue might serve as an auxiliary diagnosis marker for the occurrence of RCC.

**Table 2:** Univariate and multivariate analyses of prognostic factors and RCC survival.

	OS				DFS			
Subject characteristics	Univariate		Multivariate		Univariate		Multivariate	
	OR (95% CI)	p†	OR (95% CI)	p†	OR (95% CI)	p†	OR (95% CI)	p†
**ERβ expression high/low** *	**0.114 (0.027–0.490)**	**0.004**	**0.118 (0.015–0.916)**	**0.041**	**0.095 (0.022–0.408)**	**0.002**	**0.082 (0.011–0.636)**	**0.017**
Age	0.991 (0.961–1.021)	0.546	-	-	0.987 (0.959–1.016)	0.370	-	-
Gender – Male/Female	2.641 (0.893–7.810)	0.079	-	-	**2.937 (1.003–8.599)**	**0.049**	3.133 (0.675–14.545)	0.145
Laterality left/right	1.104 (0.477–2.556)	0.818	-	-	1.095 (0.490–2.446)	0.826	-	-
TNM stage III + IV/I + II	**5.077 (1.801–14.312)**	**0.002**	**3.595 (1.155–11.188)**	**0.027**	**5.020 (1.909–13.196)**	**0.001**	**3.290 (1.118–9.681)**	**0.031**
Grade G3 + G4/G1 + G2	**3.640 (1.230–10.766)**	**0.020**	1.441 (0.421–4.930)	0.560	**3.075 (1.146–8.251)**	**0.026**	1.070 (0.331–3.464)	0.910
Chemotherapy (no vs. yes)	2.300 (0.680–7.782)	0.180	-	-	2.141 (0.636–7.212)	0.219	-	-
Radiotherapy (no vs. yes)	1.511 (0.557–4.096)	0.417	-	-	1.789 (0.710–4.512)	0.218	-	-

* Categorized as low (≤mean) and high (>mean) was separated at ERβ >22% positivity (high) and ERβ ≤22% positivity (low).

† Analyzed with the Cox hazard regression model. Statistical significance (p<0.05) is shown in boldface. n.a.: not analyzed.

Abbrev: RCC: renal cell carcinoma, OR: odds ratio, CI: confidence interval, OS: overall survival, DFS: disease-free survival, ER: estrogen receptor.

### Effect of ERβK knockdown on cell growth

**Figure 4:**
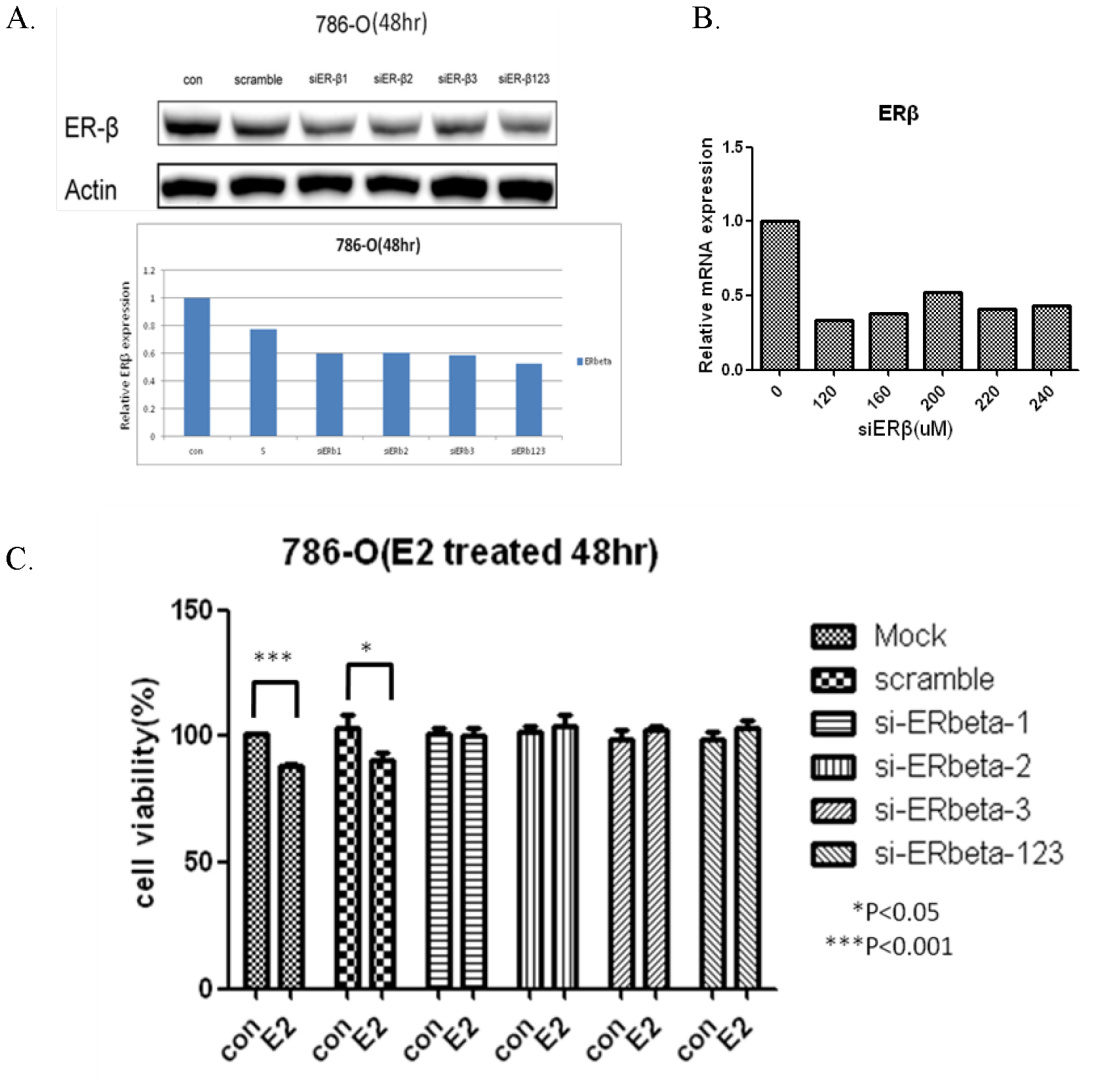
Effect of ERβ downregulation on the cell growth rate. (A) Western blot results after transfection of siERβ (siERβ1, siERβ2, siERβ3, or siERβ123) or scramble control (nonsense siRNA sequence that would not affect ERβ expression). Cells transfected with siERβ showed ERβ protein downregulation. (B) mRNA expression reduced after 786-O transfection with different concentrations of siERβ. (C) Cell growth of cells transfected with scramble control or siERβ after estrogen stimulation (10 nM) was observed by MTT assay.

ERβ expression was high in 786-O cells (Figure 1). Thus, the 786-O cell line was transfected with siRNA-ERβ to observe the phenotypes when ERβ was downregulated. siRNAx (siERβ1, siERβ2, and siERβ3) against different fragments of ERβ mRNA were designed and used for ERβ expression knockdown. Western blot analysis indicated that the three siERβ individually or in combination reduced ERβ protein expression by 40% (Figure 4A). ERβ mRNA expression was also reduced after siERβ transfection, as detected by real-time PCR (Figure 4B). In MTT assay, the results showed that after estrogen stimulation (10 nM), the cells transfected with siERβ (siERβ1, siERβ2, siERβ3, or siERβ123) to knockdown ERβ expression showed no change in cell growth rate (Figure 4C); however, the control 786-O cells showed growth rate reduction after estrogen stimulation. These results indicated that the effect of estrogen disappeared after ERβ downregulation. It was deduced that estrogen might exert its effect of reducing cell growth through ERβ activation.

### Effect of ERβ overexpression on cell growth

ERβ expression was low in A498 cells (Figure 1). Thus, the A498 cell line was used to observe the phenotypes when ERβ was overexpressed. A498 cells were transfected with pcDNA3.1-ERβ for ERβ overexpression, and pcDNA3.1 plasmid was used as control. Western blot analysis showed that cells transfected ERβ had higher ERβ expression compared with the control (Figure S1). In pcDNA3.1-ERβ, an extra V5 tag was added to distinguish extrinsic and intrinsic ERβ. Further detection of the V5 tag showed that it was only detected in the cells transfected with pcDNA3.1-ERβ, suggesting the success of transfection. The results from MTT assay showed that the growth rate of the control cells did not change after estrogen treatment. On the other hand, A498 cells overexpressing ERβ had a reduced cell growth rate (Figure S1). Combined with the results of ERβ downregulation, it is clear that estrogen affects the reduction in the cell growth rate through its interaction with ERβ.

### Effect of ERβ downregulation or overexpression on the ability of wound healing assay in RCC cell lines

The wound healing assay is one of the models for observing cell migration ability. The higher the cell migration ability, the smaller the wound area.

In the 786-O cell line, the wound area was larger in the group stimulated with estrogen (10 nM) for 24 h than in the group without estrogen stimulation (Figure S3). On the other hand, in the case of 786-O cells transfected with siERβ to reduce ERβ expression, the wound area showed no difference between the group stimulated with estrogen (10 nM) for 24 h and that without estrogen stimulation (Figure S3). At 48 h, the growth of 786-O cells had covered the wound, but cells with estrogen stimulation still showed the wound. The wound healing quantification results are shown in Figure S3B. These results suggest that estrogen stimulation (10 nM) in the 786-O cells affected the healing ability of the cells. Similarly, when ERβ expression was reduced, the effect of estrogen was diminished and the healing ability of cells was reduced.

On the other hand, the wound healing ability of A498 cells was similar with or without estrogen stimulation. Overexpressing ERβ in the low-ERβ cell line A498 and then treating the cells with estrogen for 24 and 48 h resulted in reduced wound healing ability (larger wound area) (Figure S2). The quantification is shown in Figure S2B. These results suggested that overexpression of ERβ in a low-ERβ cell line reduced the ability of the cells to heal wounds after estrogen treatment.

Combining the results of overexpression and knockdown, we deduced that estrogen must bind to ERβ to exert its effect on the wound healing ability. The results were also consistent with the MTT assay results.

### Effect of ERβ downregulation or overexpression on the ability of migration in RCC cell lines

The Transwell assay is another model to analyze cell migration ability. The difference in this assay and the wound healing assay is that in the Transwell assay, the cells must change their morphology before passing through the holes of the Transwell to the media containing 10% FBS. Thus, the Transwell assay is more representative than the wound healing assay.

**Figure 5:**
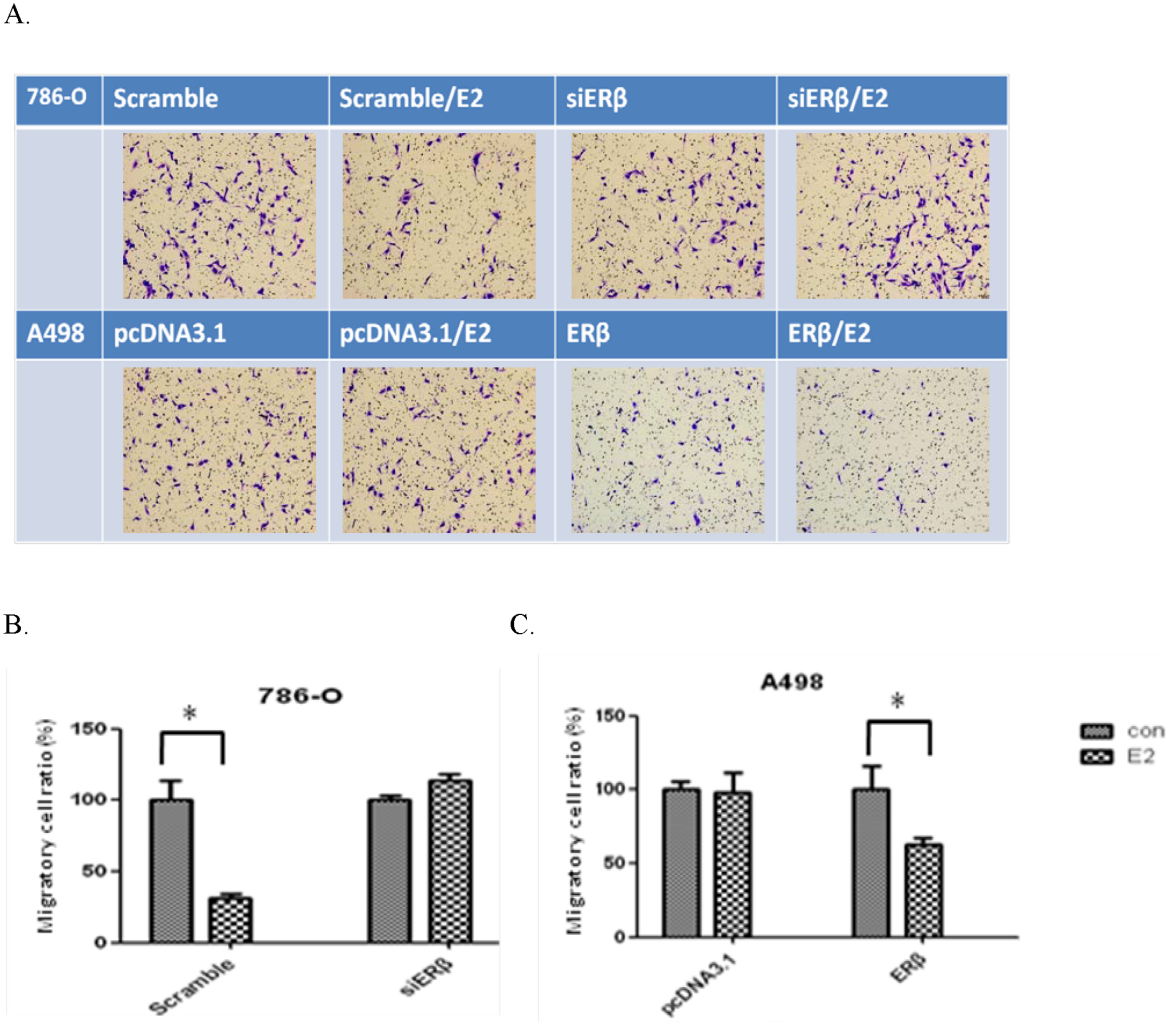
Change in migration ability after ERβ downregulation or overexpression. (A) After transfection with siERβ or pcDNA3.1-ERβ, the cells passing through the Transwell to the lower membrane were observed. (B) (C) Quantification of cells numbers that passed through the Transwell.

In 786-O cells, estrogen stimulation (10 nM) resulted in less cells passing through the membrane compared with the number without estrogen stimulation. When ERβ was knocked down in the 786-O cells, estrogen stimulation had no effect on the number of cells passing through the holes (Figure 5A), and the number of cells passing through the holes was quantified to represent the ability of cell migration (Figure 5B).

On the other hand, the numbers of cells passing through the holes were similar in A498 cells with or without estrogen (10 nM) treatment. After overexpressing ERβ in A498 cells, the cell number passing through the holes were fewer compared with the control, and estrogen stimulation (10 nM) further reduced the number (Figure 5A). The number of cells passing through the holes was quantified to represent cell migration ability (Figure 5C). These results suggest that overexpressing ERβ in low-ERβ cells reduces cell migration, and estrogen stimulation further reduces the migration ability of cells.

### Effect of ERβ downregulation or overexpression on the invasion ability of RCC cell lines

Invasion ability can also be measured by the Transwell assay. Different from the migration assay, a layer of 2% Matrigel is covered on the Transwell before cell inoculation. If the cells showed the ability of invasion, enzymes would be released to break down the structure of Matrigel. The cells are then able to crawl through the Transwell.

**Figure 6:**
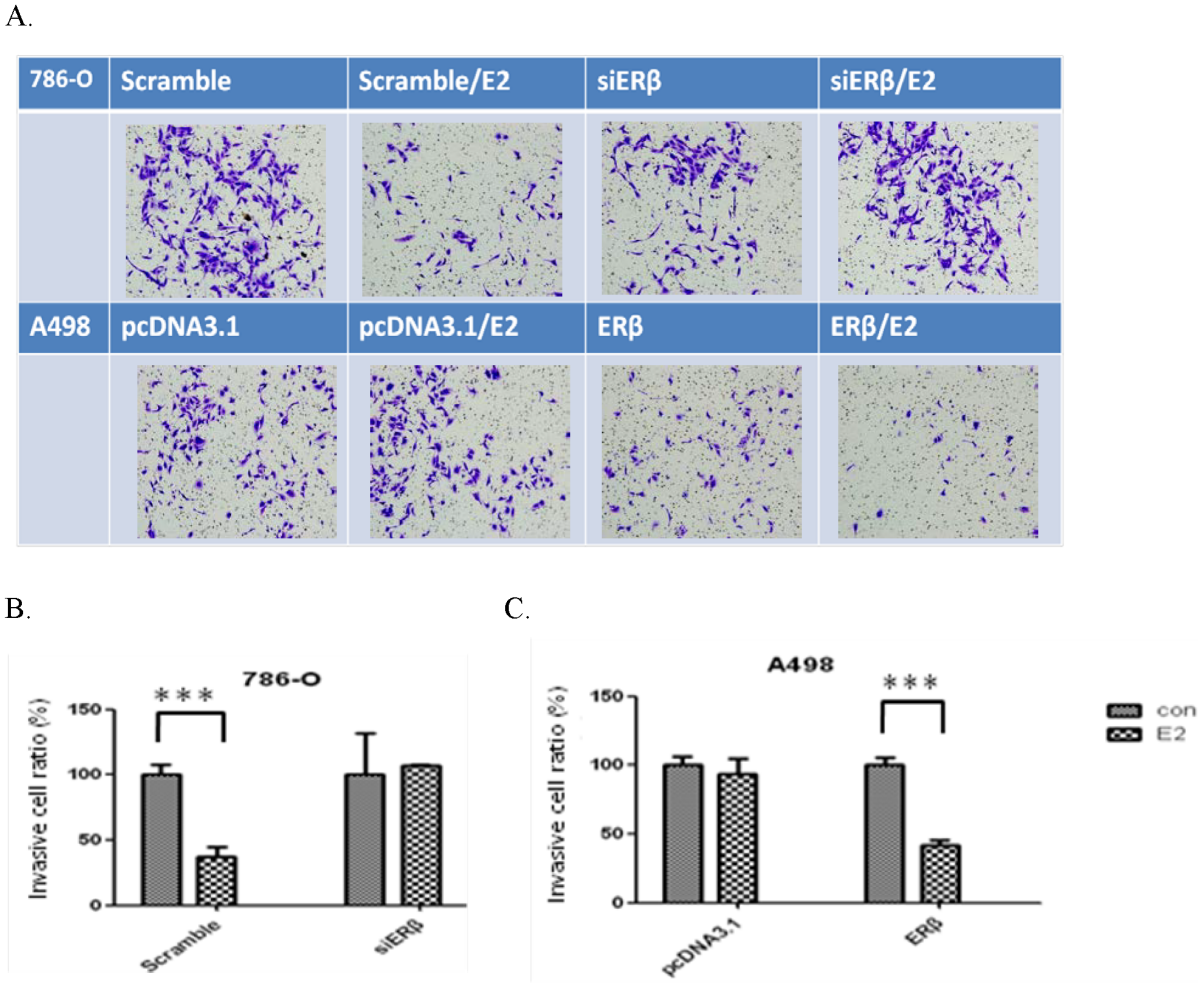
Firgure 6. Change in invasion ability after ERβ downregulation or overexpression. (A) After transfection with siERβ or pcDNA3.1-ERβ, the cells passing through the Transwell to the lower membrane were observed. (B) (C) Quantification of cell numbers that passed through the Transwell.

After stimulating the 786-O cell line with estrogen (10 nM) for 48 h, the number of cells crawling through the Transwell was reduced, indicating a decrease in invasion ability. When ERβ was reduced, the amount of cells passing through the holes showed no significant difference (Figure 6A). Overexpression of ERβ in A498 (low ERβ expression) reduced the cells' ability to invade, and estrogen stimulation further reduced cell invasion (Figure 6A). The cell number passing through the holes to the lower membrane was used to represent the invasion ability (Figure 6B, 6C).

### Changes in sub-G1 phase and cell cycle after ERβ downregulation or overexpression

After fixation with 70% ethanol and staining with propidium iodide (PI), the cell cycle of the cells in different groups was analyzed with a flow cytometer. In control 786-O cells transfected with scrambled control, estrogen stimulation (10 nM) for 48 h resulted in a 3X elevation in the percentage of cells in the sub-G1 phase. In 786-O cells transfected with siERβ, the expression of ERβ was reduced and estrogen stimulation for 48 h did not change the number of cells in sub-G1 phase (Figure S4).

On the other hand, the number of cells in the sub-G1 phase of the cell cycle showed no significant changes in A498 cells transfected with pcDNA3.1 and stimulated with estrogen (10 nM) for 48 h. With ERβ overexpression, the A498 cells showed a significant elevation in cells in the sub-G1 phase. Further estrogen stimulation (10 nM) for 48 h resulted in an increase in cells in the sub-G1 phase (Figure S5).

### Expression of EGFR signaling pathway downstream proteins after ERβ downregulation or overexpression

**Figure 7:**
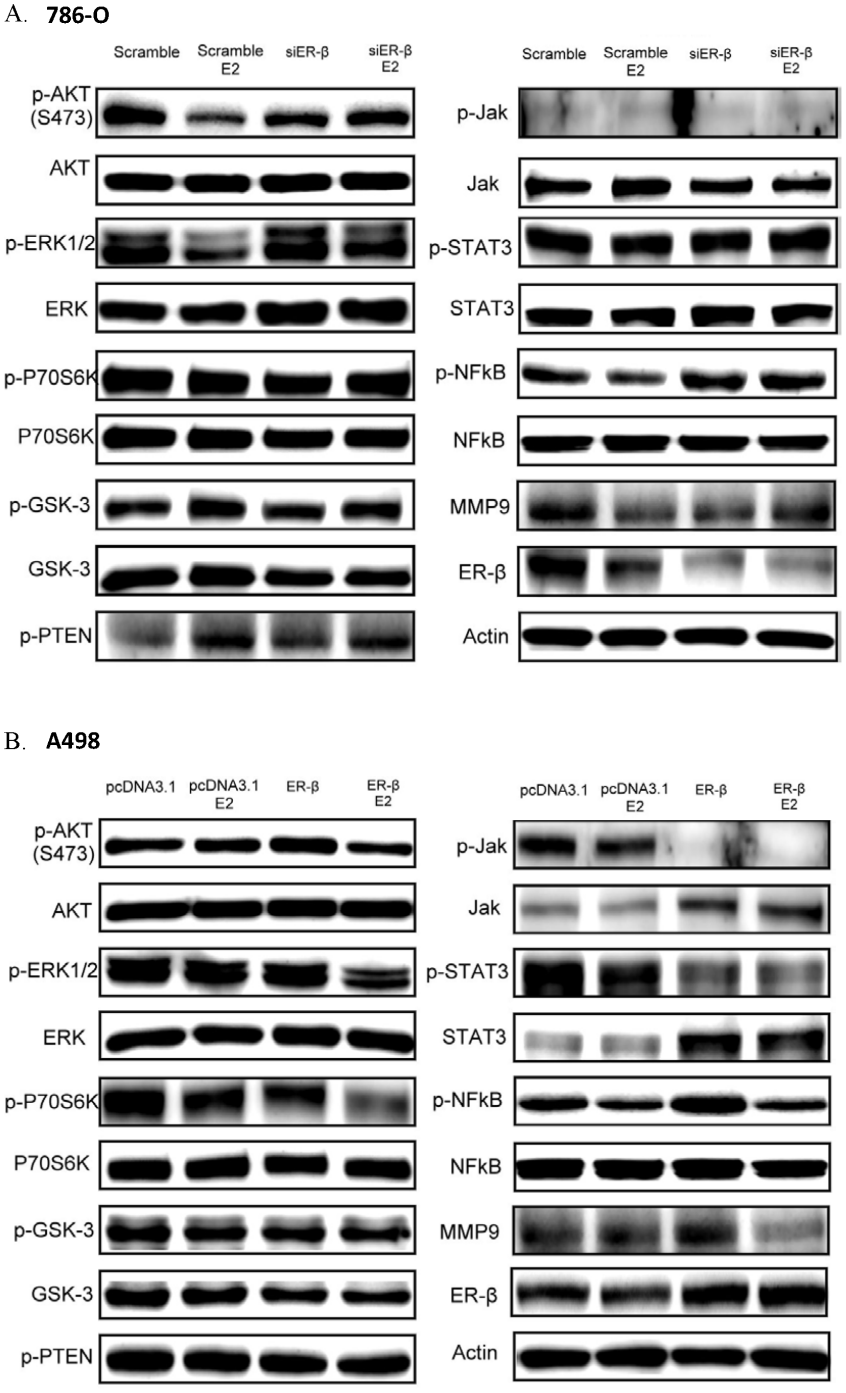
Expression of EGF signaling pathway downstream proteins in RCC cells after ERβ changed. (A) Expression of EGF signaling pathway downstream proteins in 786-O after siERβ transfection. (B) Expression of EGF signaling pathway downstream proteins in A498 after ERβ overexpression.

Previous studies showed that RCC is related to the overexpression of cell growth factors. Thus, the effects of estrogen and ERβ on the expression of growth factor-related downstream proteins were examined. In 786-O control cells, estrogen stimulation (10 nM) for 30 min resulted in the downregulation of p-AKT, p-ERK, p-NFκB, and MMP9, whereas the expression of p-GSK3 and p-TEN increased. No change was observed in p-P70S6K, p-JAK, and p-STAT3 expression. After cells were stimulated with estrogen for 30 min, the EGFR signaling pathway downstream proteins showed no changes in the siERβ transfected 786-O cells (Figure 7A).

In A498 control cells transfected with pcDNA3.1, no changes were observed in the EGFR signaling pathway downstream proteins after estrogen stimulation (10 nM) for 30 min. After ERβ overexpression, the expression of p-JAK and p-STAT3 was reduced significantly, and after estrogen stimulation (10 nM) for 30 min, the expression of p-AKT, p-ERK, p-P70S6K, p-NFκB, and MMP9 decreased. There was no change in p-GSK3 or p-TEN expression (Figure 7B).

### Expression of apoptotic signaling pathway downstream proteins after ERβ downregulation or overexpression

Flow cytometry showed that estrogen stimulation in 786-O increased the number of cells in the sub-G1 phase and that ERβ overexpression in A498 also increased cells in the sub-G1 phase. The increase in the sub-G1 phase indicates the increase in apoptosis. Thus, the expression of apoptotic signaling pathway downstream proteins was further studied.

**Figure 8:**
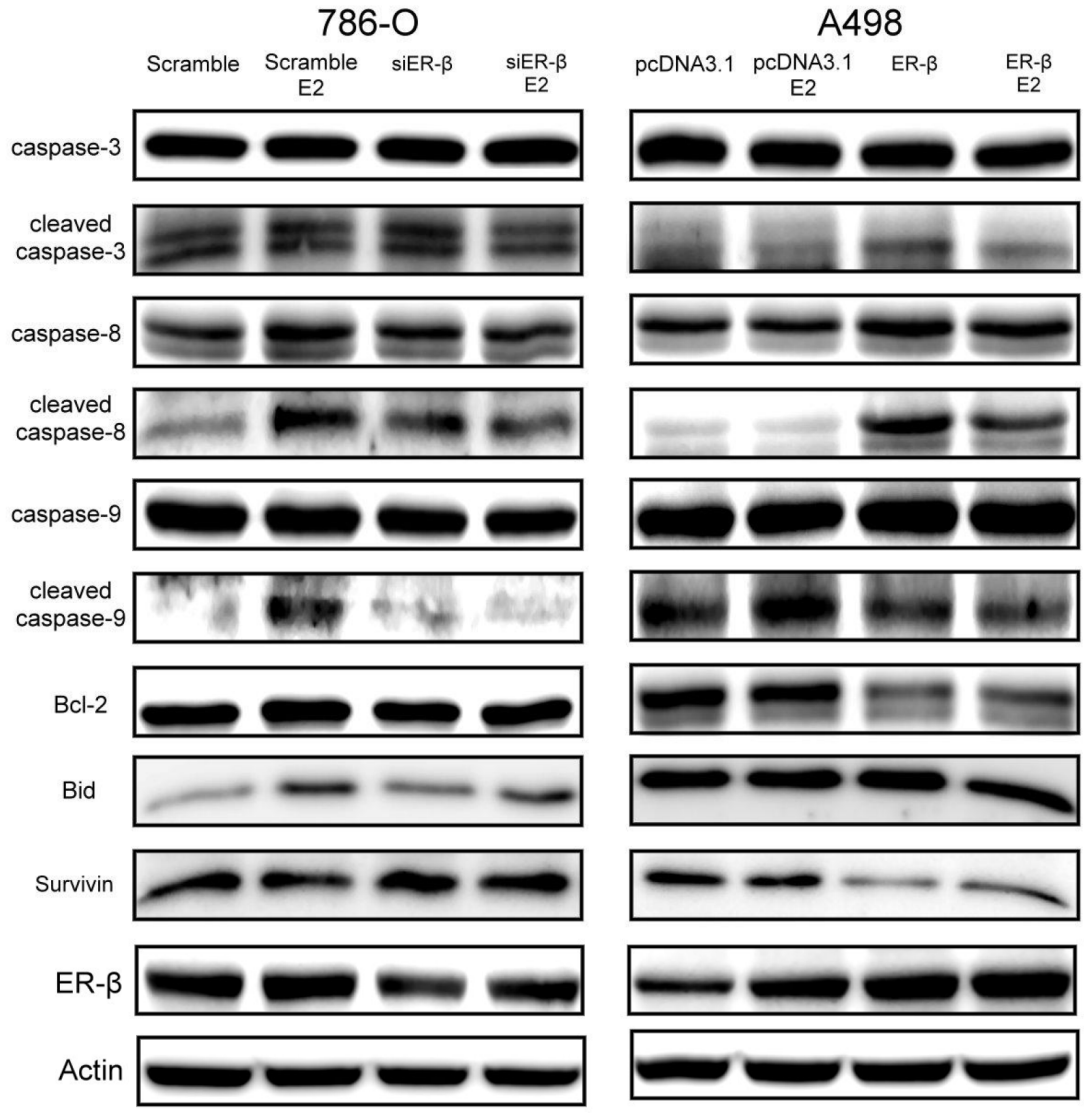
Expression of apoptotic signaling pathway downstream proteins in 786-O after ERβ downregulation and in A498 after ERβ overexpression.

In 786-O control cells transfected with scrambled control, estrogen stimulation (10 nM) for 30 min resulted in significantly increased Bid, cleaved caspase-8, and cleaved caspase-9 expression, and slightly elevated expression of cleaved caspase-3; however, survivin expression was mildly decreased and Bcl-2 expression was unchanged. After 30 min of estrogen stimulation, the apoptotic signaling pathway downstream proteins showed no significant change in siERβ-transfected 786-O cells (Figure 8).

In A498 control cells transfected with pcDNA3.1, no changes in apoptotic signaling pathway downstream proteins were found after estrogen stimulation (10 nM) for 30 min, except for slightly increased cleaved caspase-9 expression. After ERβ overexpression, the expression of Bcl-2 and survivin decreased significantly, and the expression of cleaved caspase-8 and cleaved caspase-3 increased significantly (Figure 8).

## Discussion

### ERβ expression in renal tissue

ERβ is a subtype of ER and is more extensively distributed in tissue compared with ERα. For instance, the expression of ERβ in the gastrointestinal system, lungs, and the brain is higher than that of ERα [17], [18], [19], [20]. In this study, the expression of ERβ was higher in both RCC tissue and cell lines than in breast cancer tissue and cell lines (Figure 2 and 3). In addition, ERβ showed high expression in normal renal tissue (Figure 3), the expression being higher than that in RCC tissue. These results suggested that in RCC, ERβ might play tumor suppressor role.

### Biological effects of estrogen and ERβ on RCC

Estrogen is a main female hormone involved in various cell processes, including growth, differentiation, and reproductive function. It interacts with two ERs, ERα and ERβ. After binding to the receptors, estrogen exerts its genetic or nongenetic functions through various signaling pathways. After binding to ERα, the estrogen complex promotes the transcription of growth-related factors that enhance gene expression and mitosis and promote proliferation, leading to carcinogenesis and tumor progression. Previous studies indicated that ERβ has anti-proliferative and apoptosis-inducing functions [21], [22], [19], [23]–[25]. Another study showed that estrogen activates ERβ, resulting in the elimination of cancer cells [26]. In this study, our results demonstrated that no ERα expression was observed in RCC tissue and cell lines. Thus, with estrogen stimulation, only ERβ was activated, which resulted in decreased cell growth, reduced migration and invasion ability, and increased apoptosis. After expressing ERβ in A498 cells with low ERβ expression, the abilities of cells to migrate and invade were decreased and apoptosis increased. Additional estrogen stimulation further decreased proliferation, migration, and invasion and increased apoptosis. These results showed that ERβ has the function of tumor suppression, and estrogen stimulation enhances its effect as a tumor suppressor. On the other hand, reducing ERβ expression in the high-ERβ expression cell line 786-O by si-ERβ transfection had no effect on the ability of RCC cells to proliferate, migrate, and invade, which might be due to a high tolerance for ERβ in cells with high ERβ expression. After estrogen stimulation, the function of ERβ as a tumor suppressor was also activated.

### Effect of estrogen and ERβ on the EGFR signaling pathway in RCC

Overexpression of EGFR in RCC with stimulation of epithelial growth factors activates tyrosine kinase in the cytoplasm, and phosphorylation of EGFR tyrosine kinase initiates a series of phosphorylation reactions that activate downstream gene expression, leading to uncontrolled cell proliferation and tumorigenesis. Thus, target therapy predominately focuses on the inhibition of EGFR signaling pathways. The downstream signaling pathways of EGFR are the AKT, ERK, and JAK pathways, and some studies demonstrated p-AKT overexpression in RCC [27], [28]. Previous studies also illustrated that ERβ negatively regulated HER2/HER3 and positively regulated PTEN in breast cancer, which subsequently inhibited the AKT pathway and resulted in the enhancement of tamoxifen sensitivity [23]. Thus, we further investigated the effect of estrogen and ERβ on the EGFR signaling pathway in suppression of tumor progression.

The results of this study showed that estrogen stimulation in 786-O cells with high ERβ expression resulted in negative regulation of EGFR signaling pathway downstream genes, including p-AKT, p-ERK, and p-NFκB. Among those, p-PTEN is a p-AKT inhibitor and p-GSK-3 is inhibited by p-AKT (Figure 7A). Thus, p-PTEN and p-GSK-3 regulate each other positively. When ERβ expression is reduced, additional estrogen stimulation did not affect the expression of downstream genes in the EGFR signaling pathway. Moreover, the expression of p-JAK was low in all circumstances. Thus, we deduced that the reduction in proliferation after estrogen stimulation from the estrogen-related activation of ERβ altered the expression of downstream genes in the EGFR signaling pathway.

Previous studies showed that NFκB activation increased the expression of MMP9, one of the downstream genes, and the MMP gene family is closely related to the migration and invasion of cancerous cells [29], [30]. Thus, estrogen stimulation negatively regulated the expression of MMP9, which provided the rationale for the reduction in cell migration and invasion after estrogen stimulation.

In A498 cells, which have low ERβ expression, estrogen stimulation caused no significant changes in the EGFR signaling pathway. After ERβ overexpression in A498 cells, the expression of p-JAK and its downstream gene p-STAT3 reduced significantly, whereas the protein expression of JAK and STAT3 increased. To compensate for reduced phosphorylation, JAK and STAT3 protein expression may have increased, but the level of phosphorylation was not enhanced accordingly. After estrogen stimulation, the expression of p-AKT, p-ERK, p-P70S6, p-NFκB, and MMP9 were all negatively regulated.

These results demonstrated that ERβ decreased cell proliferation through the negative regulation of the JAK pathway. After estrogen stimulation, the negative regulation of the AKT and ERK pathways resulted in reduction in cell proliferation, and negative regulation of MMP9 resulted in decreased cell migration and invasion.

### Effect of estrogen and ERβ on the apoptosis signaling pathway in RCC

Apoptosis is also known as programmed cell death. There are two steps in the process of apoptosis, which are the initiation and effector steps. The initiation step involves two pathways: the extrinsic and intrinsic pathways. The extrinsic pathway starts with the binding of receptors and ligands in the TNF receptor family. The ligands include TNF and other cytokines, the latter being secreted by immune cells such as T lymphocytes. With the assistance of the Fas-associated death domain protein (FADD), the receptors constantly collect procaspase-8 from the cytoplasm. Procaspase-8 activates itself through high density autocatalysis. Activated caspase-8 activates caspase-3 directly. Subsequently, caspase-3 interacts with its substrates for the apoptosis process. The intrinsic pathway starts with the action of tumor suppression genes, which are induced by DNA damage. Tumor suppression genes activate the expression of acting genes (*e.g.* Bax and Bad) before the effect of apoptosis starts. This leads to the release of the substances (*e.g.* cytochrome c) between the outer and inner mitochondrial membrane, and these substances act on other molecules before apoptosis [31], [32]. The apoptotic body composed of cytochrome c and Apaf-1 and procaspase-9 in the cytoplasm is the activated form of caspase-9 that cleaves caspase-3 downstream. The activated caspase-3 interacts with its substrates to promote apoptosis. In addition, activated caspase-8 lyses Bid for the formation of tBid (truncated Bid), and tBid translocates from the cytoplasm to mitochondria and induces the release of cytochrome c [33]. The main apoptosis-inhibiting proteins include the anti-apoptosis members of the Bcl-2 family (Bcl-2 and Bcl-xL). After insertion into the outer mitochondrial membrane from the cytoplasm, Bcl-2 forms a heterodimer with Bax, which inhibits the increase in mitochondria permeability caused by Bax and subsequently inhibits the release of cytochrome c from mitochondria, thus inhibiting apoptosis [34]. The other anti-apoptotic protein is survivin, which inhibits the activation of caspase-9 and then regulates apoptosis.

Previous studies have shown that in various cancer cells, ERβ induces apoptosis [24], [35], [36]. In prostate cancer, ERβ induces Bax expression, resulting in an increase in cleaved PARP and caspase-3 and apoptosis [24]. Other research has shown that activation of the EGFR signaling pathway results in increased Bcl-2 [37], leading to inhibition of apoptosis.

In our study, after estrogen stimulation, the 786-O cell line with high ERβ expression showed significant increases in caspase-8 and caspase-9, Bid activation, and some increase in caspase-3 activation; however, the expression of the anti-apoptotic protein survivin decreased slightly and no change was observed in Bcl-2 expression. In A498 cells with low ERβ expression, overexpression of ERβ resulted in a significant increase in caspase-3 and caspase-8 activation and the expression of the anti-apoptotic proteins survivin and Bcl-2 decreased evidently; however, Bid and activated caspase-9 showed no significant changes. These results suggest that ERβ reduces Bcl-2 and survivin expression and increases caspase-8 and caspase-3 activation. The lack of change in activated caspase-9 may be due to other unknown causes. After estrogen stimulation, Bid expression and caspase activation increases, promoting the process of apoptosis. These observations demonstrate that ERβ enhances apoptosis, and estrogen stimulation leads the cells toward apoptosis.

### Clinical significance of ERβ

Analysis of the tissue microarray revealed that with higher expression of ERβ, the incidence of RCC was lower. The prognosis evaluation of RCC also showed that higher expression of ERβ in RCC tissue resulted in better prognosis in RCC patients. Thus, ERβ expression has the potential of serving as the auxiliary diagnosis marker in the evaluation of RCC occurrence.

### Conclusion

The results of this study demonstrated the role of ERβ as a tumor suppressor in RCC cell lines. Estrogen stimulation activated ERβ and resulted in inhibiting cell proliferation, reducing migration and invasion, and enhancing apoptosis. Sequentially, the goal of tumor suppression would be achieved. Thus, estrogen hormone treatment might effectively activate the effect of tumor suppression through ERβ, and this might be a novel means to prevent and treat RCC. In addition, ERβ expression might serve as an auxiliary diagnostic marker in the evaluation of RCC occurrence.

## Supporting Information

Figure S1 **The effect of ERβ over-expression on cell growth rate.**(A) After over-expression with different concentration of pcDNA3.1-ERβ (0.2 µg or 0.6 µg), the expression of ERβ and V5 was observed by Western blot. (B) Cell growth of cells transfected with pcDNA3.1 or pcDNA3.1-ERβ after estrogen stimulation (10 nM) was observed by MTT assay.
(TIF)

Figure S2 **Wound healing assay in cells transfected with pcDNA3.1-ERβ.**(A) After ERβ over-expression by siERβ transfection, the effect of estrogen stimulation (10nM) was observed. (B) The quantification for wound healing ability by calculating wound area.
(TIF)

Figure S3 **Wound healing assay in cells transfected with siERβ.**(A) After ERβ down-regulation by siERβ transfection, the effect of estrogen stimulation (10nM) was observed. (B) The quantification for wound healing ability by calculating wound area.
(TIF)

Figure S4 **Changes in Sub-G1 phase and cell cycle after ERβ down-regulation.**
(TIF)

Figure S5 **Changes in Sub-G1 phase and cell cycle after ERβ overexpression.**
(TIF)
