# Supplementary figures and images for: Estrogen Inhibits Renal Cell Carcinoma Cell Progression through Estrogen Receptor-β Activation
Source: PLoS One. 2013 Feb 27;8(2):e56667. doi: 10.1371/journal.pone.0056667 (PMC3584057; doi:10.1371/journal.pone.0056667)

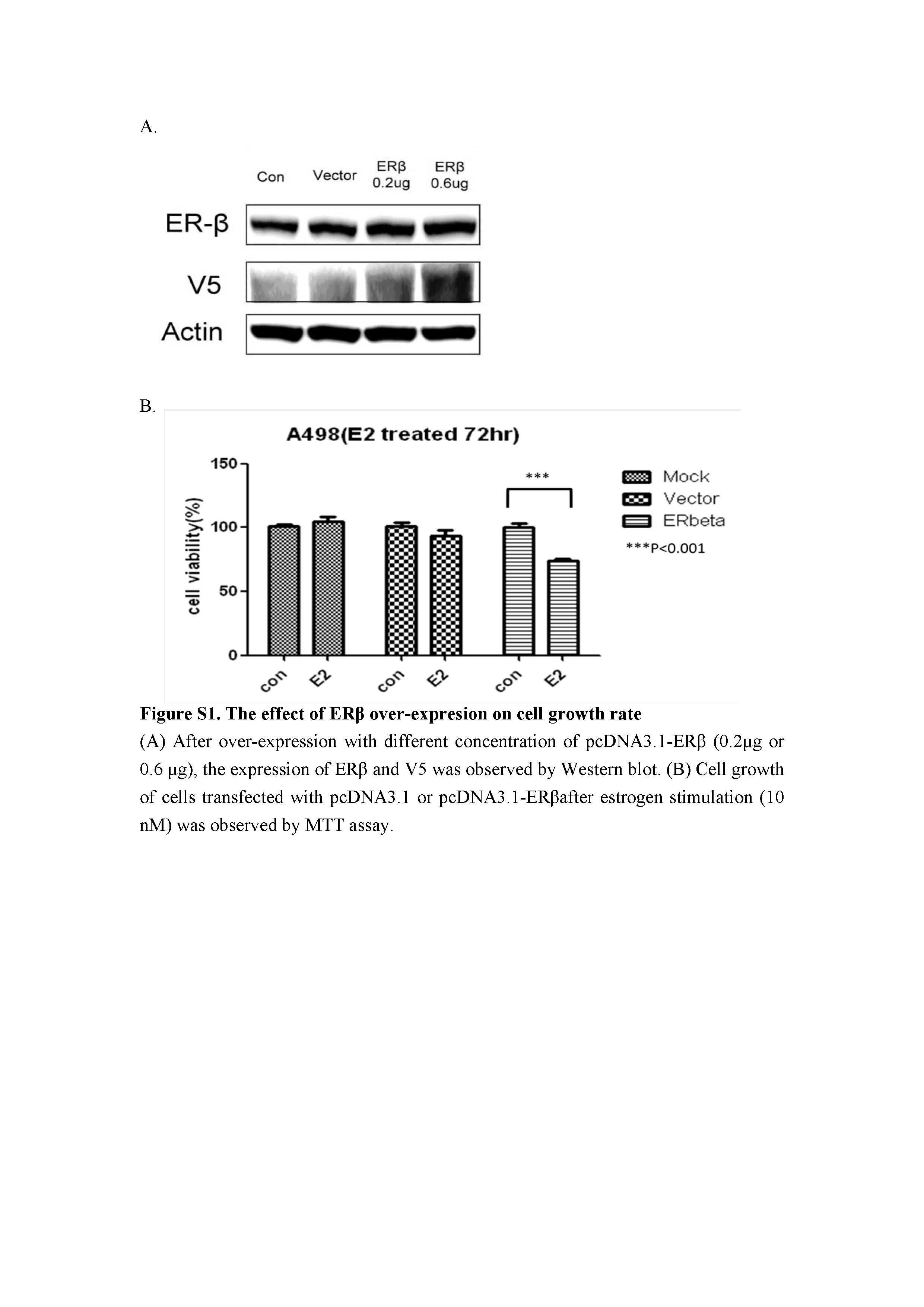

Supplement: Figure S1 — The effect of ERβ over-expression on cell growth rate.(A) After over-expression with different concentration of pcDNA3.1-ERβ (0.2 µg or 0.6 µg), the expression of ERβ and V5 was observed by Western blot. (B) Cell growth of cells transfected with pcDNA3.1 or pcDNA3.1-ERβ after estrogen stimulation (10 nM) was observed by MTT assay. (TIF) [file pone.0056667.s001.tiff]

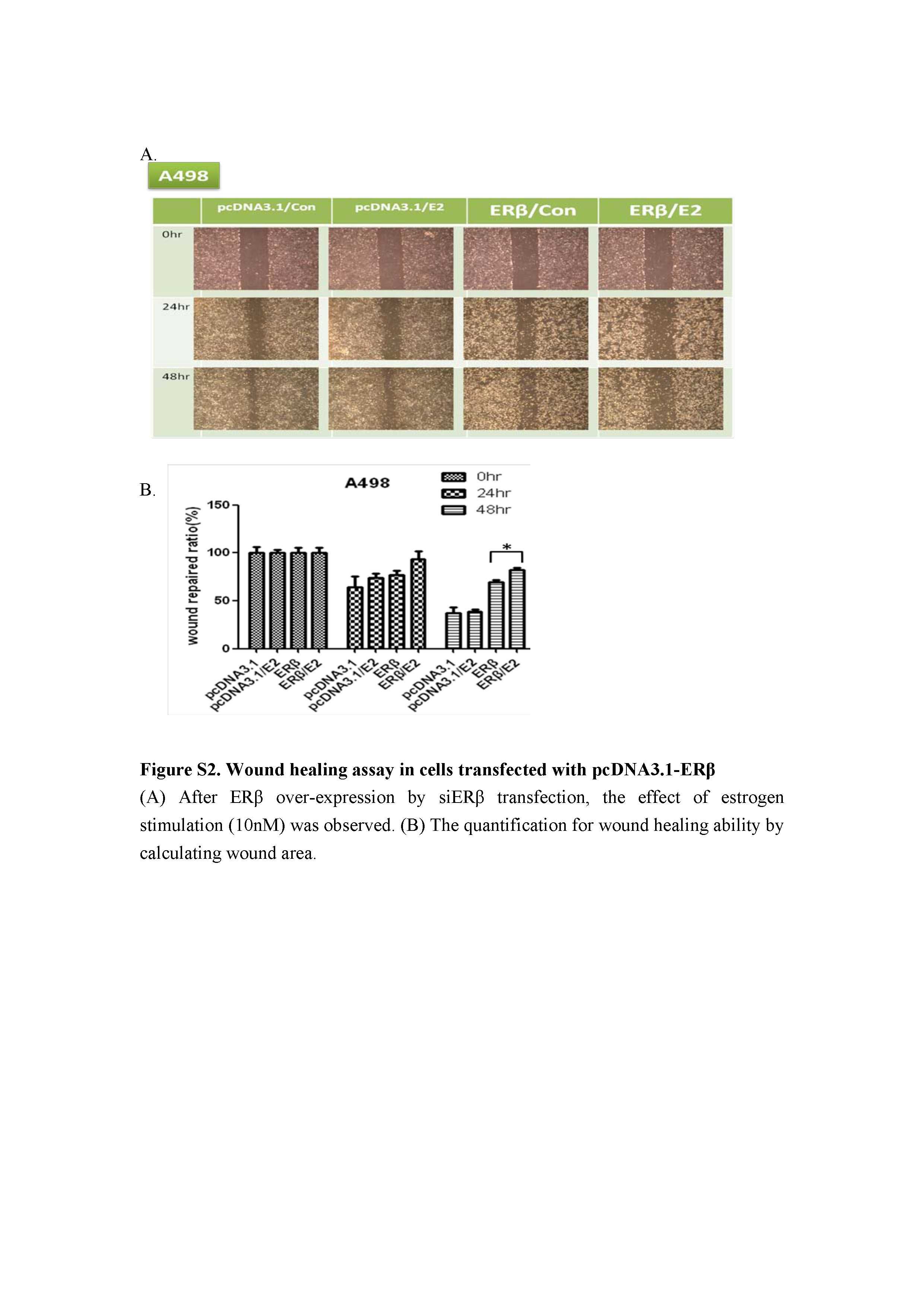

Supplement: Figure S2 — Wound healing assay in cells transfected with pcDNA3.1-ERβ.(A) After ERβ over-expression by siERβ transfection, the effect of estrogen stimulation (10nM) was observed. (B) The quantification for wound healing ability by calculating wound area. (TIF) [file pone.0056667.s002.tiff]

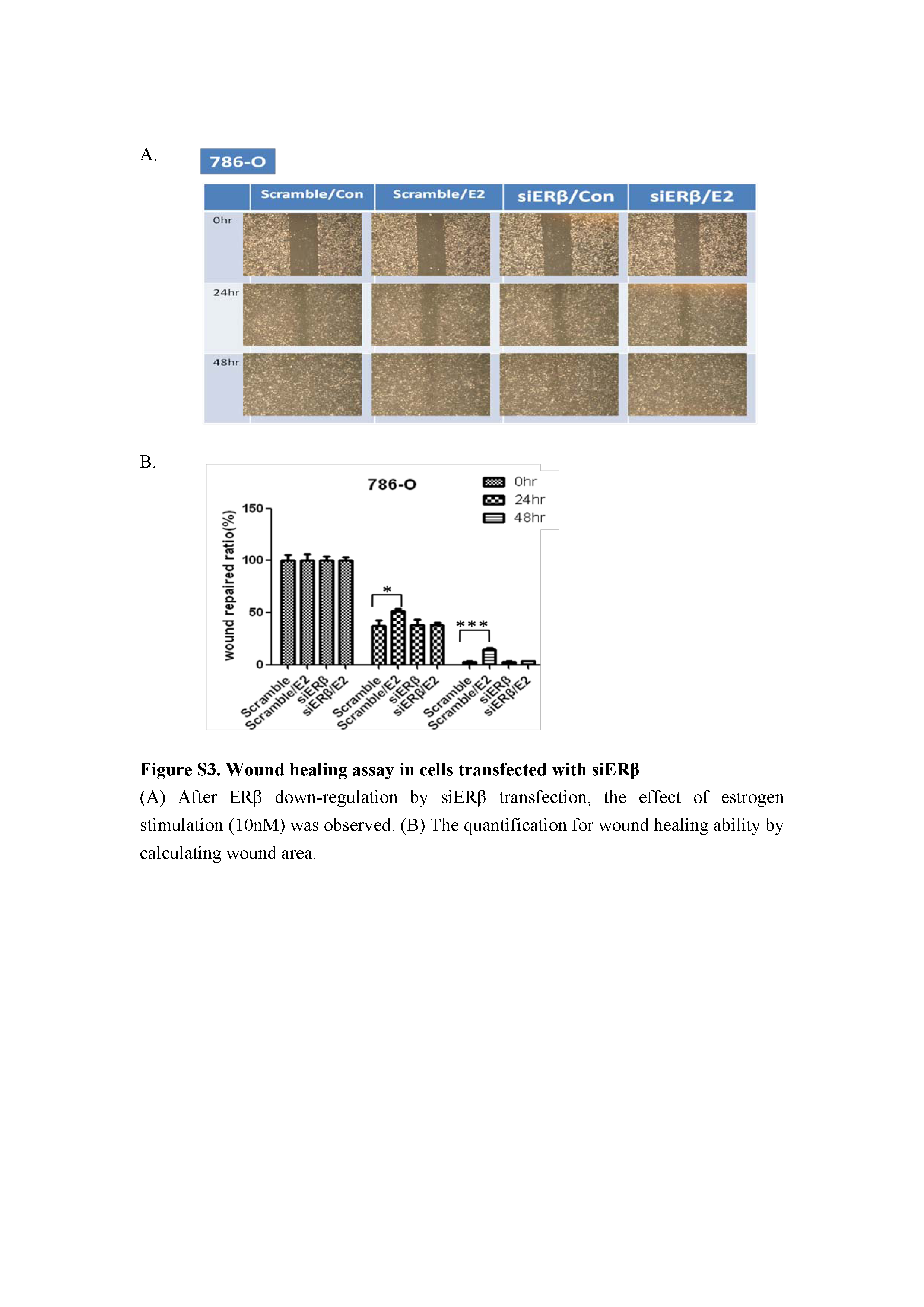

Supplement: Figure S3 — Wound healing assay in cells transfected with siERβ.(A) After ERβ down-regulation by siERβ transfection, the effect of estrogen stimulation (10nM) was observed. (B) The quantification for wound healing ability by calculating wound area. (TIF) [file pone.0056667.s003.tiff]

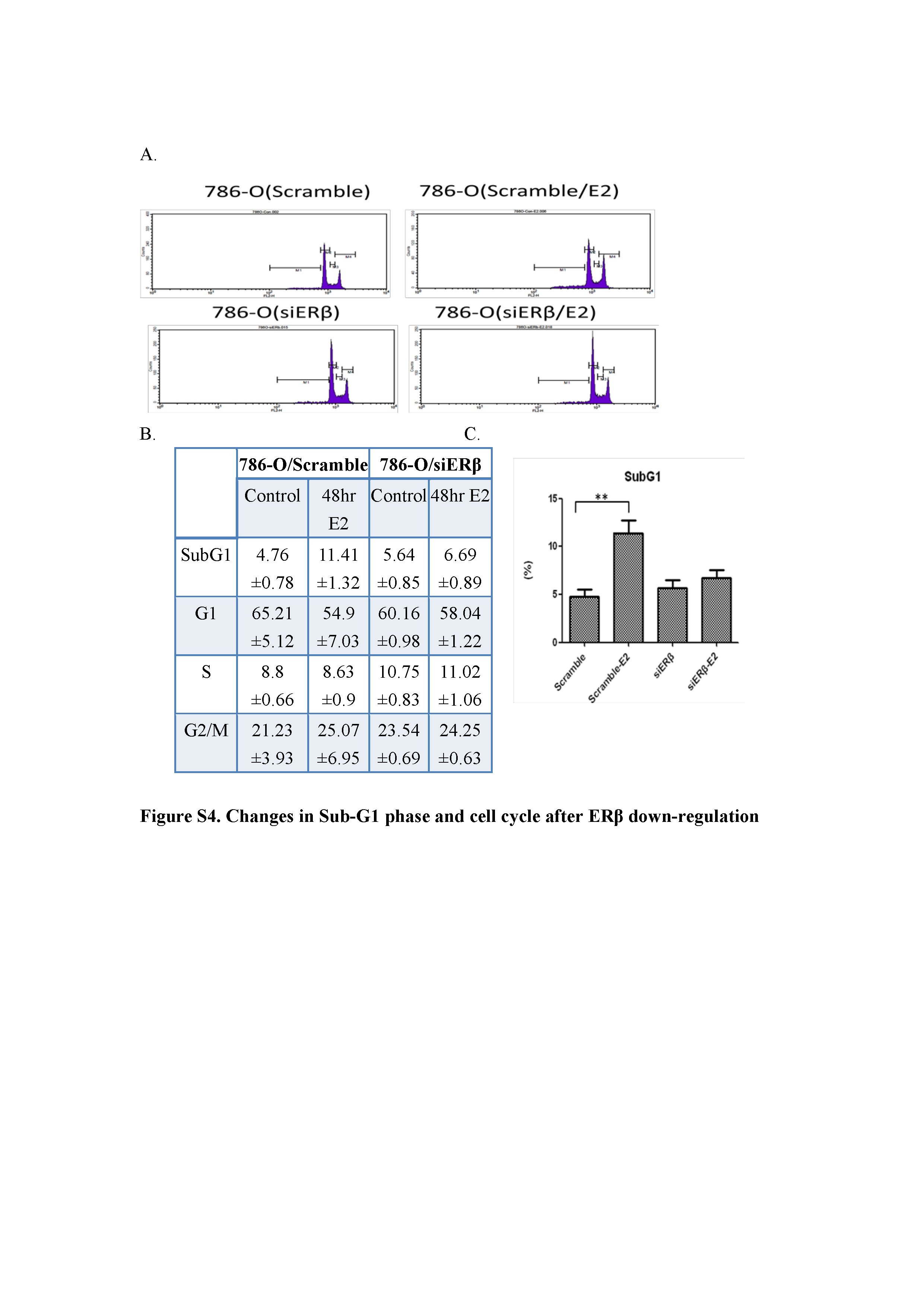

Supplement: Figure S4 — Changes in Sub-G1 phase and cell cycle after ERβ down-regulation. (TIF) [file pone.0056667.s004.tiff]

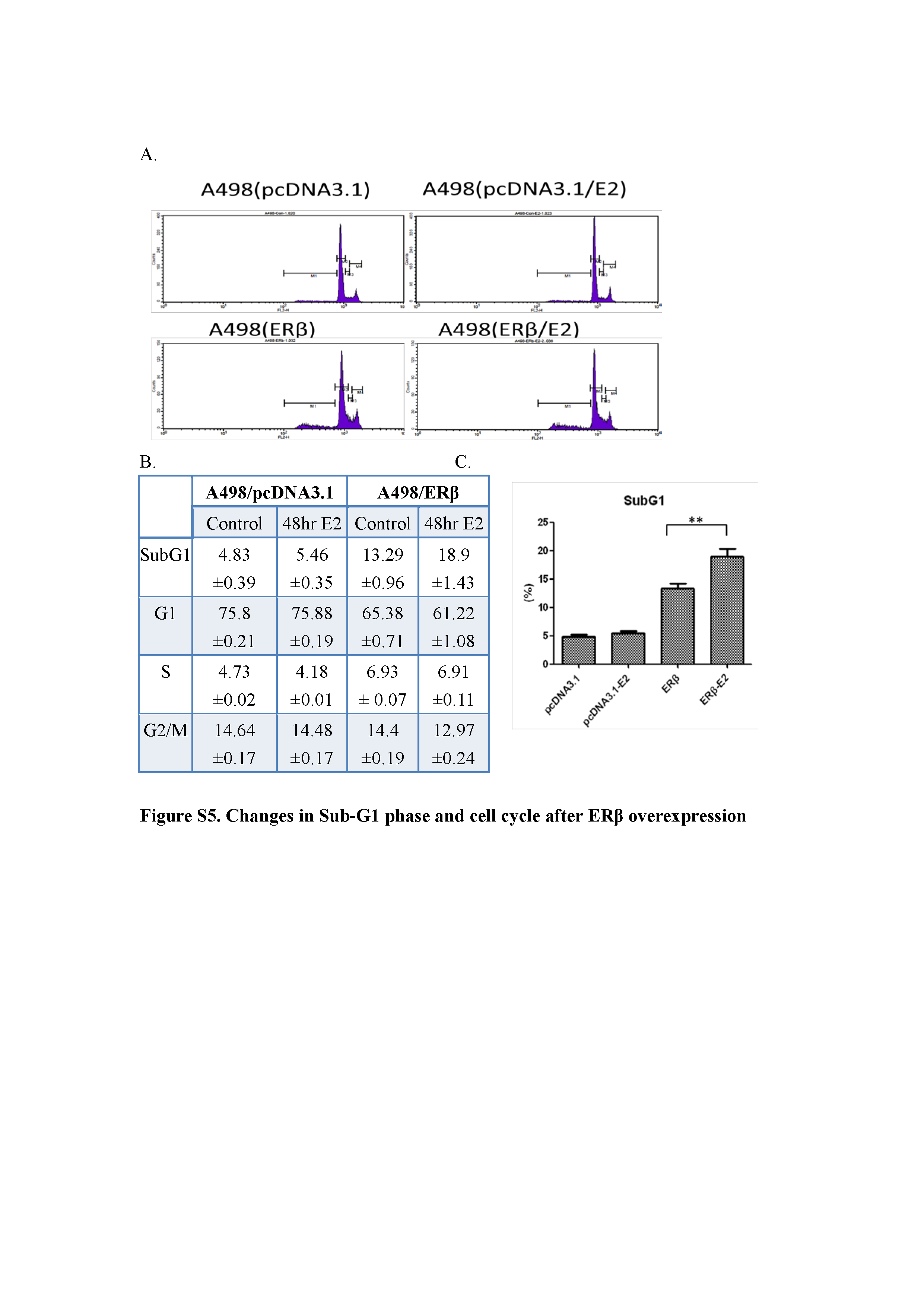

Supplement: Figure S5 — Changes in Sub-G1 phase and cell cycle after ERβ overexpression. (TIF) [file pone.0056667.s005.tiff]
